# Supplementary figures and images for: Non-destructive detection of highway hidden layer defects using a ground-penetrating radar and adaptive particle swarm support vector machine
Source: PeerJ Comput Sci. 2021 Mar 30;7:e417. doi: 10.7717/peerj-cs.417 (PMC8022505; doi:10.7717/peerj-cs.417)

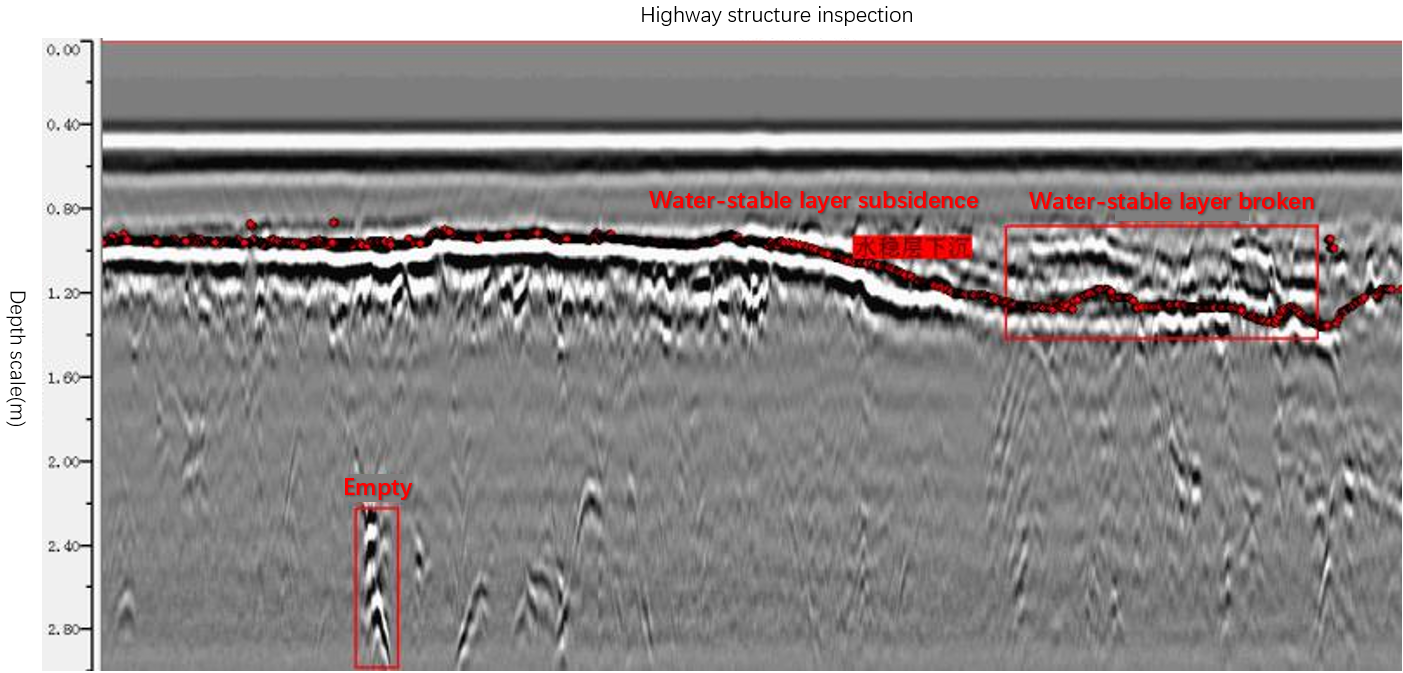

Supplement: Supplemental Information 1 [file peerj-cs-07-417-s001.png]

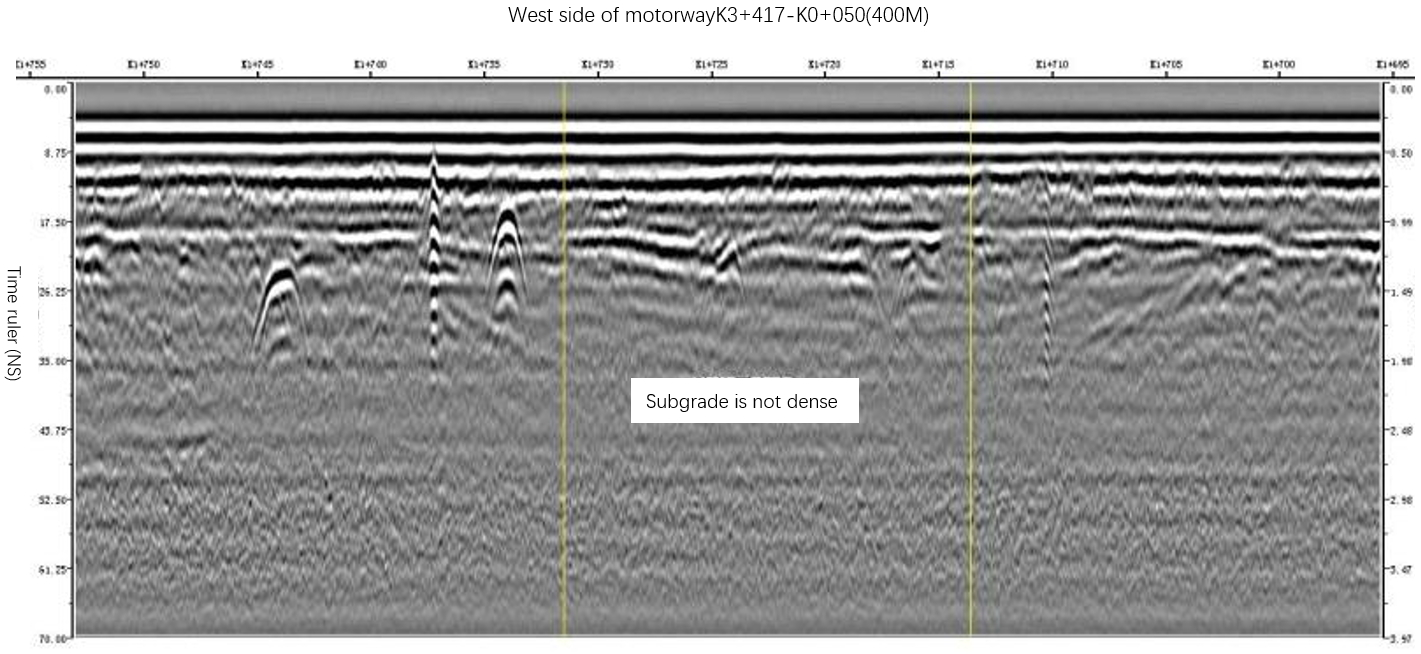

Supplement: Supplemental Information 2 [file peerj-cs-07-417-s002.png]

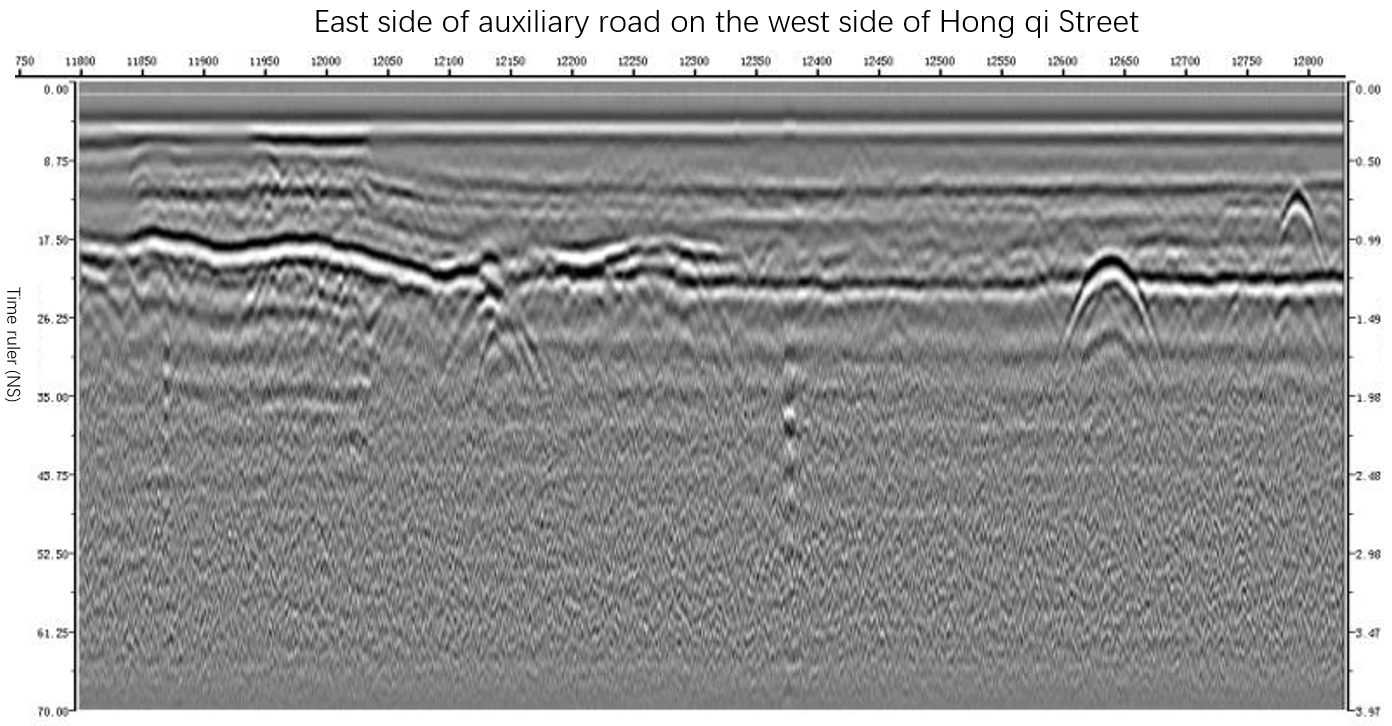

Supplement: Supplemental Information 3 [file peerj-cs-07-417-s003.png]
